# Comparison of Biosensing Methods Based on Different Isothermal Amplification Strategies: A Case Study with *Erwinia amylovora*

**DOI:** 10.3390/bios12121174

**Published:** 2022-12-15

**Authors:** Aleksandr V. Ivanov, Irina V. Safenkova, Natalia V. Drenova, Anatoly V. Zherdev, Boris B. Dzantiev

**Affiliations:** 1 A.N. Bach Institute of Biochemistry, Research Centre of Biotechnology of the Russian Academy of Sciences, Leninsky Prospect 33, 119071 Moscow, Russia; 2 All-Russian Plant Quarantine Centre, Pogranichnaya Street 32, Bykovo, 140150 Ramenskoe, Moscow Region, Russia

**Keywords:** RPA, LAMP, CRISPR/Cas, lateral flow test, nucleic acids, highly sensitive detection, isothermal amplification, fire blight

## Abstract

Isothermal amplifications allow for the highly sensitive detection of nucleic acids, bypassing the use of instrumental thermal cycling. This work aimed to carry out an experimental comparison of the four most promising techniques: recombinase polymerase amplification (RPA) and loop-mediated isothermal amplification (LAMP) coupled with lateral flow test or coupled with additional amplification based on CRISPR/Cas12a resulting from the fluorescence of the Cas12a-cleaved probe. To compare the four amplification techniques, we chose the bacterial phytopathogen *Erwinia amylovora* (causative agent of fire blight), which has a quarantine significance in many countries and possesses a serious threat to agriculture. Three genes were chosen as the targets and primers were selected for each one (two for RPA and six for LAMP). They were functionalized by labels (biotin, fluorescein) at the 5′ ends for amplicons recognition by LFT. As a result, we developed LAMP-LFT, LAMP-CRISPR/Cas, RPA-LFT, and RPA-CRISPR/Cas for *E. amylovora* detection. The detection limit was 10^4^ CFU/mL for LAMP-LFT, 10^3^ CFU/mL for LAMP-CRISPR/Cas, and 10^2^ CFU/mL for RPA-LFT and RPA-CRISPR/Cas. The results of four developed test systems were verified by qPCR on a panel of real samples. The developed assays based on RPA, LAMP, CRISPR/Cas12a, and LFT are rapid (30–55 min), user-friendly, and highly sensitive for *E. amylovora* detection. All proposed detection methods can be applied to fire blight diagnosis and effective management of this disease.

## 1. Introduction

Bacterial plant pathogens such as *Pseudomonas syringae*, pathovars *Ralstonia solanacearum*, *Erwinia amylovora*, et al. threaten the agricultural and food well-being of many countries, causing infectious outbreaks in cultivated agricultural areas, often accompanied by the need for destruction of plants and significant economic losses [1]. Since even a small number of bacterial cells present in plant material can lead to disease, detection methods are needed that detect minimal numbers of bacteria or single cells. The gold standard for the detection of microorganisms is the cultural method, which requires a significant amount of time (from several hours to several days) and a lot of labor-intensive manipulations, whereas the method does not always provide the required selectivity and sensitivity, and some pathogens are not cultivated [2,3]. Polymerase chain reaction (PCR) significantly reduces detection time and increases sensitivity and specificity of the analysis, which increases the efficiency of detection of bacterial pathogens [4,5]. However, the cyclic temperature changes of the processes—denaturation, annealing, and elongation—makes the PCR dependent on technological laboratory equipment. In addition, PCR enzymes are sensitive to inhibition by matrix components [6,7].

An alternative to PCR is isothermal amplification carried out at one temperature, which is based on the replacement of thermocycling steps with enzymes that have strand displacement activity such as *Geobacillus stearothermophilus* (Bst) DNA polymerase, *Bacillus subtilis* (Bsu) DNA polymerase I, and phi29 DNA polymerase [8,9,10]. More than ten types of isothermal amplification are known. The most common and promising are recombinase polymerase amplification (RPA) [11] and loop-mediated isothermal amplification (LAMP) [12]. Both amplification methods can be carried out at one temperature (LAMP~60 °C, RPA~37 °C) in 10–60 min depending on the recognized sequence and the type of amplification. Biosensors based on both amplifications are actively used to detect pathogenic microorganisms affecting food products and cultivated plants [13,14,15].

Another tool to improve DNA-target detection is the CRISPR (Clustered Regularly Interspaced Short Palindromic Repeats)/Cas (CRISPR associated protein) system. This system of bacterial adaptive immunity is actively used for in vitro diagnostics. Cas endonuclease in complex with guide RNA (gRNA) recognizes the target DNA (or RNA) in the sample by checking its sequence with the gRNA. Thus, due to gRNA corresponding to the pathogen target DNA sequence, CRISPR/Cas can be programmed to recognize the genetic target with high specificity. After target recognition, some class of CRISPR/Cas complex (e.g., Cas12a) acquires the ability to repeatedly cleave (trans-cleavage) any single-stranded (ss) nucleic acid; for example, Cas12a endonuclease is characterized by DNase activity [16]. In 2018, Chen et al. proposed the DETECTR (DNA Endonuclease Targeted CRISPR Trans Reporter), which is based on Cas12a for highly sensitive and specific detection of double-stranded (ds) DNA targets [17]. The DETECTR is based on the use of several reactions: target DNA amplification, recognition of amplified DNA targets by gRNA/Cas12a, trans-cleavage of ssDNA probe with an activated Cas12a in complex with target DNA, and detection of a cleaved probe. Over the past 5 years, Cas endonucleases have proven to be effective in detecting DNA and RNA targets with high selectivity [18].

The use of the CRISPR/Cas system in biosensors without additional amplifications is rather an exception since a high concentration of the target DNA (or RNA) is required to activate the Cas trans-nuclease activity. Such examples are described in detail in the review [19]; however, most designs use a combination of isothermal amplification such as RPA, LAMP, and others with CRISPR/Cas, for which extremely high sensitivity and specificity can be achieved as a result. Thus, for the highly sensitive detection of *Escherichia coli* O157:H7, the RPA–CRISPR/Cas12 system was developed [20], and LAMP–CRISPR/Cas [21]; for *Salmonella* spp.–RPA–CRISPR/Cas13 [22] and for *Campylobacter jejuni*–RPA–CRISPR/Cas12a [23]. Thus, biosensor systems based on isothermal amplifications and CRISPR/Cas are promising candidates for ultrasensitive detection of bacterial pathogens, which is confirmed by several review articles [24,25], and for the development of non-laboratory biosensor systems based on them [26,27].

**Figure 1:**
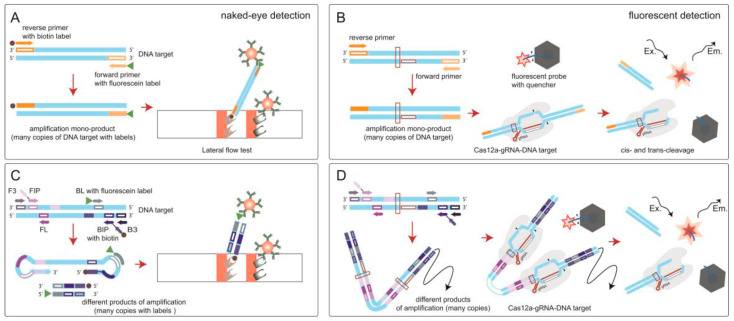
Schemes of the biosensing techniques with isothermal amplifications compared in this study using the example of *E. amylovora* detection: (**A**) RPA-LFT; (**B**) RPA-CRISPR/Cas; (**C**) LAMP-LFT; and (**D**) LAMP-CRISPR/Cas.

The advantage of biosensors based on isothermal amplification is not only highly sensitive with specificity detection, but also carries the opportunity to use simple and low-cost tools (lateral flow test (LFT), fluorescent detector) for rapid detection [28,29]. Fluorescent dyes, pH-dependent dyes, and LFTs are among the most common amplicon detection techniques. The most preferred dyes are those that are added before the reaction. This action prevents contamination because opening the tube with amplicons is excluded. pH-dependent dyes such as hydroxynaphthol blue, phenol red, cresol red, neutral red, and m-cresol purple, are successfully applied in this way and lead to end-point naked-eye visual detection of the assay results [30,31]. However, these dyes are applicable only for LAMP and unsuitable for RPA. Furthermore, widespread reagents for closed-tube detection are fluorescent dyes such as Mn^2+^ quenched calcein [32] and intercalating fluorescent dyes such as SYTO 9, SYTO 82, etc., which are optimal for real-time fluorescence detection with minimal inhibition of amplification [33,34]. For RPA, only SYBR Green and Eva Green are described as the used intercalating dyes; they are applied only as open-tube dyes, i.e., with contamination risks. Moreover, SYBR Green type dyes show greater inhibitory effects compared to SYTO dyes [33,34,35]. Thus, among fluorescent and pH-dependent dyes, a single one with equally confirmed versatility and effectiveness both for LAMP and RPA cannot be chosen. LFTs, as well as intercalating dyes, are applied after a reaction and are associated with risks of contamination. But otherwise, LFT is a simple and user-friendly tool that allows the interpretation of the results of the analysis quickly and unambiguously and does not require special equipment [36,37]. Moreover, in the study, the LFT detection provided the most identical conditions for the detection of amplicons obtained as a result of RPA and LAMP while observing additional measures to prevent contamination. For the detection of amplicons labeled with fluorescein and biotin, there are commercially available universal test strips that can be suitable for detection after RPA or LAMP. After the test strip is immersed in the sample, the amplicons move along the membrane under the action of capillary forces and are specifically recognized (streptavidin-biotin, fluorescein–antibody specific to fluorescein), resulting in staining in the test and control zones, which could be visually equipment-free estimated. The whole process takes approximately 10–15 min. Thus, the four main analytical techniques based on isothermal amplifications that are used for the rapid and sensitive detection of bacterial plant pathogens can be named: RPA-LFT, LAMP-LFT, RPA-CRISPR/Cas with fluorescence detection, and LAMP-CRISPR/Cas with fluorescence detection. However, we did not find any experimental comparison of these four techniques performed simultaneously on the same analyte. In this work, the four test systems were developed for one analyte and comparison of the effectiveness of each was carried out. Four schemes of comparable analytical techniques are shown in Figure 1. The quarantine phytopathogen *E. amylovora* causing fire blight and a serious threat to agriculture was chosen as the target object [38,39].

## 2. Materials and Methods

### 2.1. Materials

Mouse anti-fluorescein antibodies (anti-FAM) were purchased from Bialexa (Moscow, Russia). Oligonucleotides with modifications (5-carboxyfluorescein (FAM), biotin, 5-carboxyrhodamine-X [ROX], and BHQ2) were synthesized by Syntol (Moscow, Russia). DNAseI, EnGene LbCas12a, T7 RNA polymerase, RNAse inhibitor, NTP, Bst 2.0 WarmStart polymerase, an RNA cleanup kit, and a Monarch DNA gel extraction kit were purchased from NEB (Ipswich, MA, USA). Unmodified oligonucleotides, dNTP, Tersus polymerase, 100+ bp (100–1500 bp) DNA ladder, and M2 means 1 kb (250–10,000 bp) DNA ladder were obtained from Evrogen (Moscow, Russia). Protein A was produced by Imtek (Moscow, Russia). HAuCl_4_ and bovine serum albumin (BSA) were purchased from Sigma-Aldrich (St. Louis, MO, USA). The membranes as components of lateral flow strips were purchased from Advanced Microdevices (Ambala Cantt, India). All salts and organic compounds had analytical-grade purity.

### 2.2. Preparation of Bacterial Samples

The CFBP 1430 strain of *E. amylovora* (*Crataegus* sp., Lille, France, 1972) was used as a reference strain. Six additional *E. amylovora* strains, which were isolated in Russia (VNIIKR KKE3, VNIIKR TE16, VNIIKR FEa14, ACW56400, VNIIKR KE52), were used to test specificity. We also verified other pathogen bacterial species (*Dickeya solani*, *Pectobacterium atrosepticum*, *Clavibacter michiganensis* subsp. *michiganensis*, *Ralstonia solanacearum*). All bacterial strains were obtained from the bacterial collection of the All-Russian Plant Quarantine Center (Bykovo, Moscow Region, Russia); see list of the used strains in Table S1, electronic supplementary material (ESM). The bacteria cells were cultivated on Levan and King’s B agar medium was used to cultivate the bacteria cells at 25–27 °C for 48 h [40]. The bacteria were collected and suspended in 50 mM phosphate-buffered saline, pH 7.4, 100 mM NaCl (PBS). For measurement of optical density (OD), NanoDrop 2000 (Thermo Scientific, Waltham, MA, USA) was used. A concentration of about 2 × 10^9^ colony-forming unit (CFU)/mL was estimated at 0.1 OD, at a wavelength of 600 nm [41].

### 2.3. Preparing Plants for Testing

We prepared infected leaves and collected plants with and without symptoms from the local outbreak of fire blight. Apples leaves were inoculated with *E. amylovora* (CFBP 1430). For that, the leaves were cut, dipped into the bacteria suspension with about 10^9^ CFU/mL concentration, and incubated at 27 °C in humid chambers for a week until the appearance of the fire blight symptoms. The inoculated leaves were used as positive samples. Plant samples were collected in July 2022 from fire blight outbreaks located in southern regions of Russia. We collected 17 samples of apple leaves with and without symptoms.

The leaves were homogenized (150 mg) in a petri dish. For extracting total DNA, a commercial kit (PH-520, Syntol) was used.

### 2.4. qPCR for E. amylovora Detection

A commercial qPCR kit (PH-003, Syntol) intended for *E. amylovora* detection was used according to the manufacturer’s protocol, and the measurement an estimation of the cycle threshold (Ct) were carried out with the Roche Light Cycler^®^ 96 (Roche, Rotkreuz, Switzerland).

### 2.5. Primers Design

Based on the choice of genes in previous studies, three genes were selected for DNA detection of *E. amylovora*: the regulatory subunit of ATP phosphoribosyl transferase regulatory subunit (Gene ID: 8913539) (gene-1), recombinase A (Gene ID: 8914602) (gene-2), and the unannotated gene of the hypothetical AMY1267 protein (NZ_JAJTKB010000001.1) (gene-3). For the specific detection of the selected genes, both the already known and the primers proposed in this work were used (Table S2, ESM). Primers for LAMP corresponded to the sequences proposed in the article by Bühlmann et al. [42]. Primers for RPA were obtained by extending from the 5′ end of sequences proposed previously [43,44]. Chosen DNA targets were aligned using the online software BLAST and MUSCLE. To verify the dimers absent, Multiple Primer Analyzer Thermo Fisher online software was used.

### 2.6. Synthesis of the Target DNA Fragments

To produce target DNA fragments corresponding to genes 1–3, a PCR (38 cycles with denaturation: 95 °C, 30 s, annealing: 55 °C, 30 s, elongation: 72 °C, 60 s) was performed with Tersus buffer (Evrogen), 1 × Tersus polymerase, 200 µM dNTP, 500 nM of forward and reverse (sequences of PCR primers are presented in Table S2, ESM), and *E. amylovora* CFBP 1430 (5 × 10^8^ CFU/mL) as a template. PCR was performed using the BioRad T100 Thermal Cycler (Hercules, CA, USA). The obtained DNA fragments were purified by gel electrophoresis in 2% agarose in 20 mM Tris-acetate buffer with 0.2 mM EDTA, and a pH of 8.3 and a DNA gel extraction kit (Evrogen) were used. The dsDNA concentration was measured three times using NanoDrop ND-2000 (Thermo Fischer Scientific, Waltham, MA, USA).

To avoid loss of DNA during sorption on the walls of the tubes, in all experiments, we used only tubes made of certified plastic with a low binding of DNA.

### 2.7. Synthesis of gRNA for CRISPR/Cas Assay

For design of the gRNAs, CHOPCHOP (version 3) online software was used [45]. The gRNAs were obtained with in vitro transcription according to Lu and Li [46] with modifications. Briefly, the reaction was performed for 3 h at 37 °C; the reaction mix (100 µL) contained 40 mM of Tris-HCl, pH of 8.0, 2 mM of spermidine, 20 mM of MgCl_2_, 1.25 mM of each NTP, 10 mM of DTT, 2.5 u/µL of T7 RNA polymerase, 0.5 u/µL of RNAse inhibitor, and 2 µM of DNA template. Then, 3 U DNAseI and 10 µL of DNAseI buffer were added and incubated for 30 min at 37 °C (this repeated stage). For RNAs purification, the RNA cleanup kit (NEB) was used.

### 2.8. Preparation of the Lateral Flow Test Strips

For test strips, plastic supports with the nitrocellulose membrane (CNPC-12), glass fiber membrane for conjugate (PT R5), adsorbed pad (AP045), and sample pad (GFB R4) were used. All membranes were produced by Advanced Microdevices (Ambala Cantt, India). The gold nanoparticles (GNPs) as a label for test strips were synthesized by the citrate reduction method [47], and the conjugate of GNPs and anti-FAM antibodies (Bialexa) were synthesized according to Safenkova et al. [48] (details of both syntheses are given Section S2, ESM). Streptavidin and protein A (Imtech) were applied to the test and control zones. An IsoFlow Dispenser (Imagene Technology, St. Lebanon, NH, USA) was applied to disperse the reagents. The dispensing, assembly, and cutting of test strips were described in detail in the previously presented protocol [48].

### 2.9. RPA with LFT

An RPA basic kit (TwistDx, Cambridge, UK) was applied for the amplification. Briefly, 300 nM bio- and FAM-labeled primers were mixed with the rehydration buffer. Then, 10 µL of sample with a DNA template was added. The lyophilized pellet from the basic RPA kit (TwistDx, Cambridge, UK) was dissolved in the mixture (final volume was equaled 50 µL). At the final stage, 14 mM magnesium acetate was added; the reaction was performed for 20 min at 39 °C in the T100 Thermal Cycler (BioRad, Hercules, CA, USA). After the reaction, 5 µL of the RPA mixture solution was inserted into 65 µL of PBS (sample for LFT assay). To avoid contamination when opening the tube with the reaction mixture, it was always carefully controlled so that all the reaction liquid was at the bottom of the tube. In addition, the detection was never carried out in the room where electrophoresis was performed. These rules were followed for all types of amplifications.

The LFT was dipped into the sample. After 10 min, the qualitative results were estimated visually, and the visual detection limit was set as the target concentration at the weakest-colored signal in the test zone. All test strips were scanned by Canon Scanner (Tokyo, Japan) for the quantification of the results. The color intensities of the test zones were estimated with TL120 (Nonlinear Dynamics, Newcastle upon Tyne, UK). A signal that was below 2 arbitrary units (arb.u.) was considered as the eye-visible limit and was set as negative. Each measurement was conducted in duplicate.

### 2.10. LAMP with LFT

LAMP was performed using Bst 2.0 WarmStart (NEB) polymerase (320 U/mL), LAMP buffer (NEB, Ipswich, MA, USA), and three pairs of primers—F3 and B3 (200 nM), FIB and BIP (1.6 μM)—and an additional pair—FL and BL (400 nM). The reaction was carried out in the presence of dNTP (1.4 mM each) and 8 mM magnesium sulfate. Then, 10 µL of samples with template were added to the LAMP mixture (final volume was equaled 20 µL). The reaction carried out at 65 °C for 30 min in the T100 Thermal Cycler (BioRad, Hercules, CA, USA). After the reaction, 5 µL of the LAMP mixture solution was added to 65 µL of PBS and used as a sample for LFT as described above (see RPA with LFT). For DNA fragments of the target gene, the LAMP products were also visualized with electrophoresis in 1% agarose gel in 20 mM Tris-acetate buffer with 0.2 mM EDTA.

### 2.11. RPA-CRISPR/Cas and LAMP-CRISPR/Cas

For both assays, RPA and LAMP were performed as described above (see RPA with LFT and LAMP with LFT). NEB2.1 buffer with 30 nM of gRNA and 30 nM of LbCas12a were mixed and incubated at 25 °C for 10 min. Then, 500 nM of ROX-dT15-BHQ2 probe (Table S2, ESM) was added. The reaction of the cleavage began upon the addition of the 2 µL sample with RPA or the LAMP-amplified DNA target at 37 °C. The total volume of the reaction was 30 μL. The ROX fluorescence (λ_ex_: 578 nm; λ_em_: 604 nm) was measured every 30 s by Light Cycler 96 (Roche). Three standard deviations of the null sample signal were used for detection limit estimation.

### 2.12. The Spearman Correlation

To compare the data obtained with five different methods for *E. amylovora* detection, the Spearman correlation using GraphPad Prism 9.0.2 (GraphPad Software, San Diego, CA, USA) was performed. For correlation, we used the data from real samples and artificially inoculated samples. Spearman correlation coefficients were obtained for each pair of methods (qPCR, RPA-LFT, LAMP-LFT, RPA-CRISPR/Cas, and LAMP-CRISPR/Cas).

## 3. Results and Discussion

### 3.1. LAMP and RPA Detection of the DNA Fragments of Target Genes

For RPA and LAMP, we selected three genes for which the efficiency and specificity of PCR as an RPA prototype or LAMP have been previously shown. Each gene is presented as a single copy in the genome. Moreover, we evaluated the probability of alternative DNA forms (Z-DNA, G-quadruplex, etc.) or rare local conformational elements (hairpin, loops with unpaired nucleotides, etc.) in these genes (the features of target genes are presented in Table S3). The genes tend to have no non-canonic structures. The selected gene targets for LAMP and RPA have different lengths and GC content. However, the mechanism of two amplifications is so different that even within the same gene it is impossible for RPA and LAMP to obtain the same amplicons [49]; therefore, given the same copy number and proven efficiency of primers, different gene localization is not a significant factor.

DNA fragments of target genes 1–3 were obtained from *E. amylovora* CFBP 1430 using the PCR primers shown in Table S2 and purified after agarose gel electrophoresis (Figure S2). Using the obtained fragments as templates, LAMP and RPA were tested.

**Figure 2:**
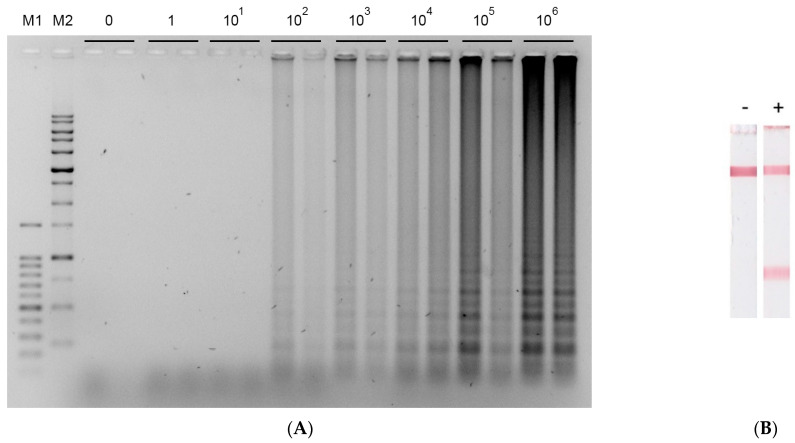
Visualization of LAMP products after amplification of phosphoribosyl transferase gene (gene-1) (**A**) after electrophoresis in 1% agarose gel: 0, 10, 100, 1000, 10^4^, 10^5^, 10^6^ copies per reaction, M1 means 100+ bp (100–1500 bp) DNA ladder, M2 means 1 kb (250–10,000 bp) DNA ladder; (**B**) using LFT recognized FAM/biotin labeled amplicons (BL-primer labeled with FAM, BIP primer labeled with biotin) “-” LAMP-LFT without the DNA template, “+” LAMP-LFT in the presence of 10^4^ copies of the DNA template.

To implement LAMP, we used six primers previously proposed in the work [42]; forward inner primer (FIP), backward inner primer (BIP), forward outer primer (F3), and backward outer primer (B3) were required for the reaction to proceed, and two loop primers (forward loop primer (FL) and backward loop primer (BL)) were able to improve the amplification efficiency. To carry out LAMP with the selected set of primers, we used a temperature of 65 °C for 30 min, which was previously selected by Bühlmann et al. [42]. As a result, LAMP with electrophoretic detection showed a sensitivity equal to 100 copies in the reaction (Figure 2A). A sharp change in the amount of the resulting amplification product indicates a narrow range of quantitatively recognizable concentrations. Thus, the visualization of LAMP products in agarose gel suggests rather a qualitative assessment.

After confirmation of the amplicon formation with the selected primers, primers with FAM or biotin labels were obtained, which were necessary for the detection of amplicons by LFT. In LAMP, FAM and biotin can be introduced into the amplicons using four types of labeled primers (FIP, BIP, FL, BL). Two outer primers (F3 and B3) were necessary for strand displacement of the characteristic FIP- and BIP-related elongation product and were not of interest for labeling. We tested 12 primer/label combinations in which two of the four primers were unlabeled, one included the FAM, and one included biotin. This strategy reduces the number of labeled primers on the test strip that compete with the labeled amplicons. All possible primer/label combinations were tested using 10^4^ copies per reaction as the concentration to produce a large number of amplicons. The test strip recognized FAM/biotin-labeled amplicons due to streptavidin in the test zone, protein A in the control zone, and a conjugate of gold nanoparticles with antibodies specific to FAM (see Figure 1C). As a result, BL-primer labeled with FAM and BIP primer labeled with biotin provided the maximum signal during the detection of amplicons by LFT (Figure 2B); for the other combinations, the coloration of the test zone was either absent or manifested much weaker. Accordingly, the combination of labeled primers (BL-FAM, BIP-biotin) can be further used to test LAMP-LFT on total bacterial DNA samples, artificially contaminated extracts of plant samples, healthy and naturally infected samples.

**Figure 3:**
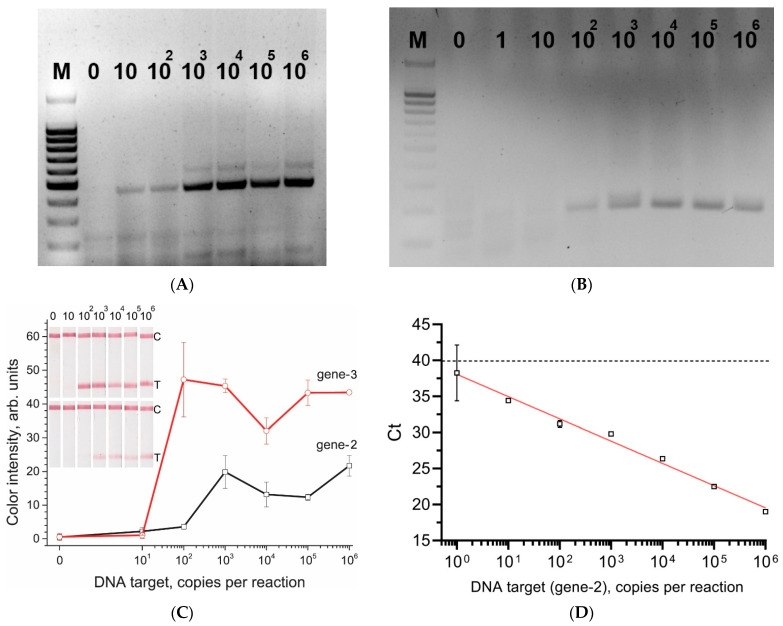
Results of amplification of the DNA fragments of gene-2 and gene-3. (**A**) RPA visualization of gene-2 amplicons on 2% agarose gel after electrophoresis; (**B**) RPA imaging of gene-3 amplicons on 2% agarose gel after electrophoresis; (**C**) test strips after RPA-LFT (the number of DNA copies in the reaction is indicated above the strips; 0 is the negative control, C means control zone, T means test zone) and the corresponding concentration dependences of the intensity of the test zones on a number of DNA copies; (**D**) Calibration plot of qPCR for DNA fragment of gene-3. Dash line indicates the limit cycle for reliable detection of gene-3.

To implement RPA, we chose target genes that were previously successfully used for PCR diagnostics: the recombinase A gene [44] (gene-2) and chromosomal gene sequence coding for a hypothetical protein (systematic i.d. ‘AMY1267’) [43] (gene-3). RPA primers were designed based on the PCR primers by extending them to 33 nucleotides in accordance with the recommended lengths [11], and fluorescein and biotin labels were introduced at the 5′ ends of the forward and reverse primers, respectively (see Table S2, ESM). For RPA, a wide temperature range (37–42 °C) provides a similar sensitivity [50], so we chose a temperature of 39 °C as recommended by the manufacturer (TwistDx). RPA with electrophoretic detection showed a sensitivity equal to 10 (gene-2, Figure 3A) and 100 copies (gene-3, Figure 3B) in the reaction. However, for gene-2, in addition to the target product, reaction by-products were visualized by electrophoresis (Figure 3A). The test strips used the same assembly as for the detection of amplicons after LAMP (see Figure 1A,C). As a result, the sensitivity of RPA-LFT was 100 copies per reaction for both genes (Figure 3C). These values correspond to the sensitivity of PCR performed for the same genes (Figure 3D). Figure 3B shows that the signal differs from the background starting from 100 copies (negative control corresponded to 35 cycles). As in the case with LAMP, a rather sharp increase in the signal was observed when moving to higher concentrations. At the same time, for gene-3, more intense coloration of the test zone was observed for all tested concentrations than for the gene-2. Accordingly, primers for the amplification of gene-3 were chosen for further experiments.

### 3.2. Comparison of Four Amplification Strategies for the Detection of E. amylovora Cells

To compare amplification techniques, a series of dilutions of bacterial cells in PBS was made from 1 to 10^7^ cells per sample. After that, LAMP or RPA was performed for each dilution, and one part of the reaction mixture was used immediately for the detection of amplicons with test strips (RPA-LFT, LAMP-LFT), and the second part for CRISPR/Cas recognition and detection of the fluorescence of the probe (ROX-15T-BHQ2) cleaved with activated Cas12a (RPA-CRISPR/Cas, LAMP-CRISPR/Cas).

Conducting the RPA-LFT and LAMP-LFT (see Figure 1A,C) was actually no different from the DNA fragment of the target genes described above. Whereas RPA-CRISPR/Cas and LAMP-CRISPR/Cas techniques required additional components and reactions, for the detection signal after CRISPR/Cas, a probe labeled with a ROX was used to eliminate the signal of FAM-labeled primers and amplicons. Guide RNAs were designed based on target-gene sequences with two main conditions: neighboring position to protospacer adjacent motif (PAM), which was firstly identified in the DNA target by CRISPR/Cas12a, and did not include the primer zone (see details in Table S2, ESM). The gRNAs synthesized by reverse transcription were used to obtain a complex with Cas12a.

**Figure 4:**
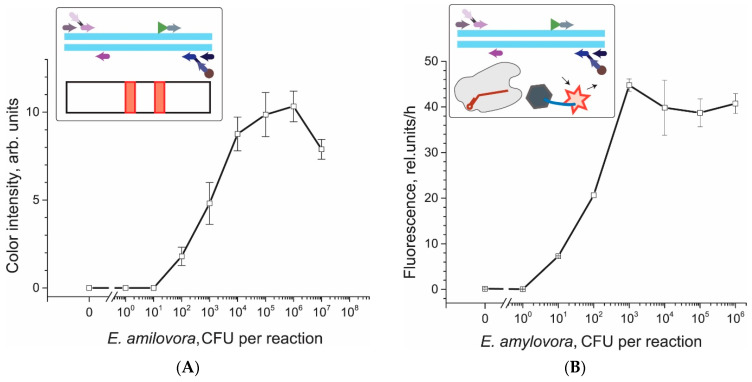

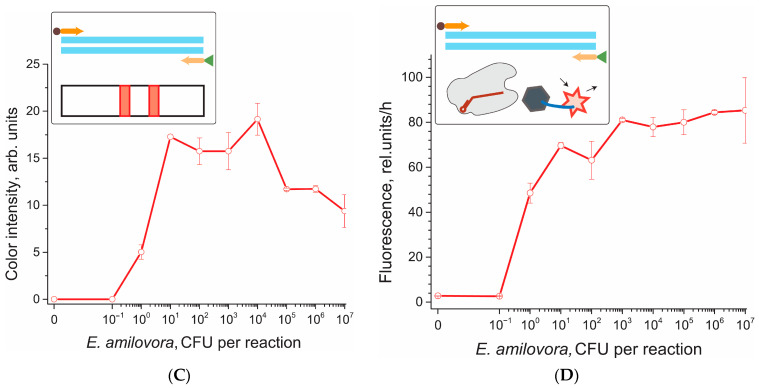
Concentration dependences for *E. amylovora* cells used for detection by different amplification techniques: (**A**) LAMP-LFT; (**B**) LAMP-CRISPR/Cas; (**C**) RPA-LFT; and (**D**) RPA-CRISPR/Cas.

As a result, for all amplification techniques, there was no signal for negative samples and concentration dependences were observed (Figure 4). Using the three-sigma method, cut-off values were set for the RPA-CRISPR/Cas (3.18 rel. units) and LAMP-CRISPR/Cas (0.34 rel. units) to determine positive samples (for RPA-LFT and LAMP-LFT, cut-off values accorded to minimal coloration for reliable visual detection and were equal to 2 arb. units for both cases). The results presented in Figure 4 show that the limit of detection was 100 CFU/reaction (reaction volume 10 µL) or 1 × 10^4^ CFU/mL for LAMP-LFT, 10 CFU/reaction or 1 × 10^3^ CFU/mL for LAMP-CRISPR/Cas, 1 CFU/reaction for gene-3 (reaction volume 10 µL) or 100 CFU/mL for RPA-LFT, and 1 CFU/reaction for gene-3 or 100 CFU/mL for RPA-CRISPR/Cas. Therefore, for both schemes with PRA, extremely high sensitivity was shown. For RPA-CRISPR/Cas, the signal for 1 CFU/reaction was only approx. 40% below the maximum, and for RPA-LFT, it was approx. 72% below the maximum. If the sensitivity of the isothermal amplification assay provides the detection of single cells (RPA-LFT), then the addition of extra amplification of CRISPR/Cas did not change the detection limit but increased the difference between the detected signal and the background. If the sensitivity of the assay did not correspond to single cells (LAMP-LFT), then the detection limit can be reduced by adding additional amplification of CRISPR/Cas.

Only one article describes the scheme using RPA for the detection of *E. amylovora*-RPA-CRISPR/Cas with a FAM-BHQ probe [51]. The authors used phosphoribosyl transferase as the target gene and showed a detection limit of 10^3^ CFU/mL. The limit of detection for PCR diagnostics based on the same gene-3 and the same but shortened primers was 1 × 10^3^ CFU/mL [52]. Two systems have been published for LAMP, both with fluorescent detection and a detection limit of (1) 10 CFU/reaction or the equivalent of 1.2 × 10^4^ CFU/mL for detection of the same phosphoribosyl transferase target gene [42], and (2) 10 CFU/reaction or 10^4^ CFU/mL for histidine-tRNA ligase gene detection (EAMY_RS32025) [53]. However, in the article comparing LAMP and PCR [52], LAMP-based primers from [53] and qPCR assays showed high specificity for *E. amylovora* and were able to detect up to ×10^3^ CFU/mL. Regarding immunoassays [41,51], all test systems based on isothermal amplifications turned out to be expectedly more sensitive, by 2–4 orders of magnitude depending on the type of test system.

Obviously, not for all target genes and not for all bacterial pathogens, the limit of detection in RPA-LFT and RPA-CRISPR/Cas is equal to single cells. This result depends on many factors, including the representation of the selected gene in cells. The results indicate that, in terms of sensitivity, the addition of a CRISPR/Cas stage is not always required, and may complicate (add new components) and affect the duration of (additional 20 min) the analysis, without leading to a decrease in the detection limit. However, for the scheme with LAMP, we note the justification for using the CRISPR/Cas stage.

The uniqueness of the selected DNA fragments was shown earlier based on bioinformatics analysis, as well as the high specificity of the detection of *E. amylovora* by PCR and LAMP [42,43,44]. However, to confirm this, we tested several common bacterial pathogens *D. solani*, *P. atrosepticum*, *C. michiganensis* subsp. *michiganensis*, and *R. solanacearum*. All bacteria showed no cross-reactivity in PCR, LAMP, and RPA (Figure S3, ESM). For *E. amylovora* belonging to other isolates (VNIIKR KKE 3, VNIIKR FEa14, ACW56400, VNIIKR TE 16, VNIIKR KE 52), significant positive signals were shown in PCR, LAMP, and RPA.

### 3.3. Comparison of Four Amplification Strategies in the Analysis of Healthy, Artificially Infected, and Diseased Plants

To validate the four proposed amplification assays, we used healthy and artificially infected plant samples, as well as a panel of samples collected from a location of natural outbreak of fire blight. The total DNA was isolated from each sample, after which five tests were performed: PCR, LAMP-LFT, RPA-LFT, LAMP-CRISPR/Cas, and RPA-CRISP/Cas. Each sample was analyzed by each method in at least two repetitions.

**Figure 5:**
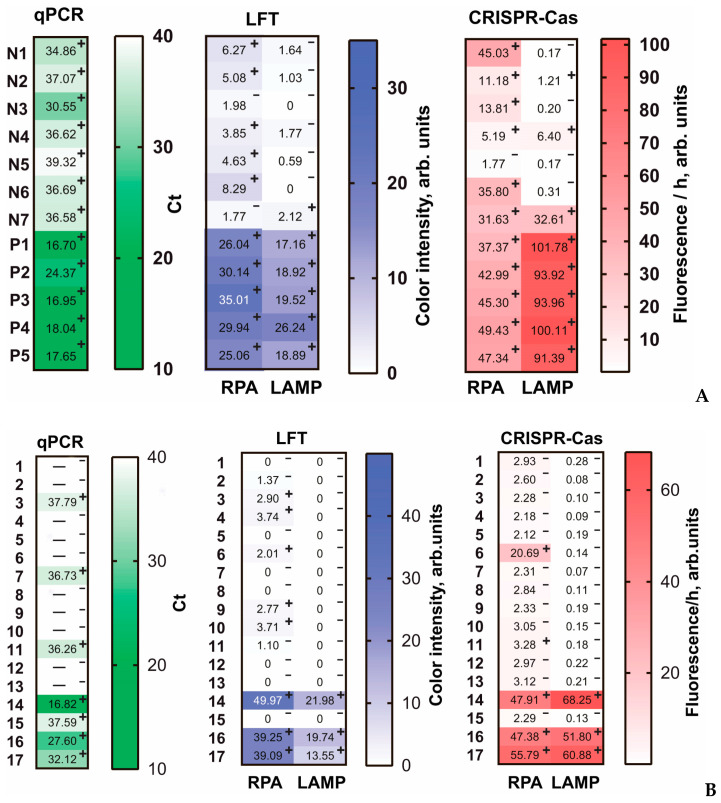
Detection of *E. amylovora* in plant leaves by qPCR, RPA-LFT, LAMP-LFT, RPA-CRISPR/Cas, and LAMP-CRISPR/Cas. The results for each sample are presented as distinct squares in the heat maps. (**A**) artificially contaminated extracts of plant samples and (**B**) real samples (dash lines in qPCR row mean no detectable signal). Positive samples are marked with “+”, negative samples are marked with “-” (cut-off for qPCR: 40; RPA-LFT and LAMP-LFT: 2.0; RPA-CRISPR/Cas: 3.18; LAMP-CRISPR/Cas: 0.34).

For five artificially contaminated samples (P1–P5), all five methods showed a strong positive signal (PCR: Ct > 25) (Figure 5A). For plants that were not artificially infected and were probably naturally infected, PCR showed a weakly positive response, which was best confirmed by RPA-CRISPR/Cas (confirmed for N1–N4, N6–N7). RPA-LFT was not confirmed by PCR in all cases (N3 and N7 were negative). LAMP-LFT showed a negative result for all weakly positive samples (N1–N7) detected by PCR. LAMP-CRISPR/Cas confirmed a positive signal for three weakly positive samples (N2, N4, N7).

For real samples consisting of 17 samples collected in July 2022 in outbreaks of fire blight in the southern regions of Russia, all five methods showed a strong positive signal for three samples (14, 16, 17) (Figure 5B). In addition, RPA-CRISPR/Cas identified sample #6 as strongly positive. However, this result has not been confirmed by any other method and we assume that was a false positive due to contamination. In total, RPA-LFT, LAMP-LFT, RPA-CRISPR/Cas, and LAMP-CRIPSR/Cas were in full agreement for positive samples determined by PCR with Ct > 33. Weakly positive samples with Ct > 33 most successfully confirmed the RPA-based test systems. To evaluate the correlation of the obtained results, we carried out the Spearman correlation for data presented in Figure 5 and calculated Spearman correlation coefficients (r_s_). The correlations of qPCR data with four developed methods were negative and high (r_s_ < −0.75) or moderate (−0.75 < r_s_ < −0.5); the Spearman correlation coefficients are presented in Figure S4. Between the four developed methods, the correlation analysis showed a high (r_s_ > 0.75) or moderate (0.5 < r_s_ < 0.75) positive correlation. When comparing methods, it is important that, unlike PCR, RPA-LFT and RPA-CRISPR/Cas are much faster to implement (30 and 45 min, respectively), do not require complex equipment such as a thermal cycler, and a 37 °C heater (RPA-LFT, RPA-CRISPR/Cas) and a simple fluorescent detector (RPA-CRISPR/Cas) are enough. For LAMP-LFT and LAMP-CRISPR/Cas, the analysis time was 40 and 55 min, respectively; a 37 and 60 °C heaters (LAMP-LFT, LAMP-CRISPR/Cas) and a simple fluorescent detector (LAMP-CRISPR/Cas) were required. In addition, it is important to consider that LAMP-LFT or LAMP-CRISPR/Cas, with the detection method involving open-tubes techniques, very often provide false positive results caused by carryover contamination [54,55]. This shortcoming can be prevented by implementing closed-tube detection devices or carefully separated places for sample preparation and for analysis [56,57,58]. Therefore, the comparison showed the advantages of RPA-based strategies for *E. amylovora* detection, which allows for a recommendation of RPA-based methods to rapidly and conveniently monitor fire blight-infected plants.

The additional potential of the RPA-LFT and PRA-CRISPR/Cas systems is the ability to carry out amplification directly in the sample without preliminary extraction of total DNA. This is confirmed in several works on the diagnosis of plant pathogens, including the diagnosis of *E. amylovora* [51,59]. In this work, the approach without sample extraction was not tested in order to be able to compare the same DNA samples by both PCR and isothermal test systems.

## 4. Conclusions

Three new diagnostic test systems have been developed for the detection of *E. amylovora*: LAMP-LFT, LAMP-CRISPR/Cas, RPA-LFT, and the test system of the RPA-CRISPR/Cas, which was first proposed for the unannotated gene of the hypothetical AMY1267 protein. For the first time, using the developed test systems as an example, we checked the comparison of four amplification strategies on the same analyte, and also verified the results using the qPCR method. Comparison of RPA and LAMP followed by amplicon detection with LFT and CRISPR/Cas with fluorescence detection showed that LAMP-CRISPR/Cas was superior in sensitivity to LAMP-LFT, RPA-LFT, and RPA-CRISPR/Cas, which detected single cells in the reaction and was superior in sensitivity to LAMP-CRISPR/Cas. The developed RPA-LFT and RPA-CRISPR/Cas demonstrated the lowest detection limit for *E. amylovora* relative to previously published test systems. Thus, we have shown that the inclusion of additional CRISPR/Cas amplification in the assay does not always lead to an increase in sensitivity. The isothermal amplification can be sufficient to provide high sensitivity and specificity. In this regard, the development of double detection systems such as RPA-CRISPR/Cas and LAMP-CRISPR/Cas in the absence of direct experimental verification of the system with only one amplification solution does not seem to be a rational solution.

The testing of the four developed test systems on real samples showed that all the proposed detection methods can be applied to the fire blight diagnosis and effective management to prevent the damage to agricultural crops. All proposed test systems were able to detect *E. amylovora* in plants with and without symptoms of the disease.

## Data Availability

The data presented in this study are available on request from the corresponding author.

## Supplementary Materials

The following supporting information can be downloaded at: https://www.mdpi.com/article/10.3390/bios12121174/s1, Table S1. Bacteria used in the study, Table S2. Sequences of DNA used in the study, Table S3. Characterization of target genes, Table S4. Comparison of test strips after testing LAMP products after amplification of phosphoribosyl transferase gene (gene-1) using all combinations of labeled primers, Figure S1. Distributions of hydrodynamic diameters of gold nanoparticles, their conjugates with anti-FAM antibodies, and conjugate buffer obtained through DLS, Figure S2. Fragments of target genes after electrophoresis in 2% agarose gel, Figure S3. Testing bacterial pathogens with RPA, LAMP, and PCR, Figure S4. Spearman correlation coefficients for data obtained at the testing samples from plant leaves by qPCR, RPA-LFT, LAMP-LFT, RPA-CRISPR/Cas, and LAMP-CRISPR/Cas.
