# Supplementary material for: Comparison of Biosensing Methods Based on Different Isothermal Amplification Strategies: A Case Study with Erwinia amylovora
Source: Biosensors (Basel). 2022 Dec 15;12(12):1174. doi: 10.3390/bios12121174 (PMC9776058; doi:10.3390/bios12121174)
Supplement: Supplementary file 1 [file biosensors-12-01174-s001.zip › biosensors-2012652-supplementary.pdf]

biosensors

Supplementary Materials

Article

Comparison of Biosensing Methods Based on Different Isothermal Amplification Strategies: A Case Study with *Erwinia amylovora*

Aleksandr V. Ivanov [1], Irina V. Safenkova [1], Natalia V. Drenova [2], Anatoly V. Zherdev [1] and Boris B. Dzantiev [1,*]

[1] A.N. Bach Institute of Biochemistry, Research Centre of Biotechnology of the Russian Academy of Sciences, Leninsky Prospect 33, 119071 Moscow, Russia
[2] All-Russian Plant Quarantine Centre, Pogranichnaya Street 32, Bykovo, Ramenskoe, 140150 Moscow Region, Russia
* Correspondence: dzantiev@inbi.ras.ru; Tel.: +749-5954-3142

Contents

Section 1. Using bacteria and oligonucleotides in the study 2

Section 2. Preparation of gold nanoparticles and conjugate of gold nanoparticles with antibodies 6

Section 3. Characterization of DNA fragments of target genes obtained from *E. amylovora* 7

Section 4. Comparison of test strips after testing LAMP products using all combination of labeled primers 8

Section 5. Cross-reactivity testing 9

Section 6. Spearman correlations of data for *E. amylovora* detection by five methods 10

Section 1. Using bacteria and oligonucleotides in the study

Table S1. Bacteria used in the study.

No	Bacterial specie	Strain	Geographic origin	Year	Source
1	*Dickeya solani*	DSM 28711	The Netherlands	before 2010	DSMZ
2	*Ralstonia solanacearum*	NCPPB 2316	Australia	1970	National Collection of Plant Pathogenic Bacteria
3	*Pectobacterium atrosepticum*	DSM 18077	The Netherlands	2007	DSMZ
4	*Clavibacter michiganensis* subsp. *michiganensis*	CM4761	Hungary	2007	AOBC PPSCD
5	*Erwinia amylovora*	VNIIKR KKE 3	Russia, Krasnodar	2017	VNIIKR
6		VNIIKR FEa14	Poland	2018	Kaliningrad IVL
7		ACW56400	Switzerland	2007	ACW
8		VNIIKR TE 16	Russia, Tambov	2010	VNIIKR
9		VNIIKR KE 52	Russia, Kaliningrad	2020	Kaliningrad IVL
10		CFBP 1430	France	1972	CFBP

Table S2. Sequences of DNA used in the study.

Method	Gene	Name	Sequence 5′-3′	Source
LAMP	ATP phosphoribosyl transferase regulatory subunit (gene-1)	F3	TCAAGATCGTGTGGCTATG	(Bühlmann, Pothier et al. 2013)
		B3	CTAAAAACCGGGGCAAAC	
		FIP	ACGATTCTACCCTTCCTGTCTACTTCTCTGGGGTTTCAGTC	
		BIP	ATGTCACCTGATTCTACAGCCGCAATCATTCATGGTTCTGGAC	
		FL	ATTAGCGGCCCGACCAA	
		BL	GTTAAGATGGCATGCAGA	
		BIP (Bio)	Bio–ATGTCACCTGATTCTACAGCCGCAATCATTCATGGTTCTGGAC	Proposed in this study
		BL (FAM)	Bio-GTTAAGATGGCATGCAGA	
PCR		F	TAGATAAATCAATTAAAGGCCA	
		R	GTCTACCTTAAATCCTTACTGC	
gRNA template		Gene-1 sgRNA F	TTTTTTTTTAATACGACTCACTATAGGTAATTTCTACTAAGTGTAGATGGTGATAAAACCTTTGAATT	
		Gene-1 sgRNA R	AATTCAAAGGTTTTATCACCATCTACACTTAGTAGAAATTACCTATAGTGAGTCGTATTAAAAAAAAA	
RPA	recombinase A (gene-2)	recA_1 RPA F	FAM-GTTATTGCCGCCGCACAGCGTAAAGGTAAG	Proposed in this study
		recA_1c RPA R	Bio-GATACGGCGAATATCCAGACGGACAGAG	
gRNA template		Gene-2 sgRNA F	TTTTTTAATACGACTCACTATAGGTAATTTCTACTAAGTGTAGATTCAACCAGATCCGTATGAAAAT	
		Gene-2 sgRNA R	ATTTTCATACGGATCTGGTTGAATCTACACTTAGTAGAAATTACCTATAGTGAGTCGTATTAAAAAA	
PCR		recA_1	CCGCACAGCGTAAAGGTAAG	(Gehring and Geider 2012)

		recA_1c	GAATATCCAGACGGACAGAG	
RPA	AMY1267 (gene-3)	hpEaF RPA F	FAM-GCTCTCATTGCCGTGGAGACCGATCTTTTA	Proposed in this study
		hpEaF RPA R	Bio-TTATAACAAAAGTTTCTCCGCCCTACGAT	
PCR		hpEaF	CCGTGGAGACCGATCTTTT	(Gottsberger 2010)
		hpEaR	AAGTTTCTCCGCCCTACGAT	
gRNA template		Gene-3 sgRNA F	TTTTTTAATACGACTCACTATAGGTAATTTCTACTAAGTGTAGATAGAGAGGCAGCATTCGACGAAC	Proposed in this study
		Gene-3 sgRNA R	GTTCGTCGAATGCTGCCTCTCTATCTACACTTAGTAGAAATTACCTATAGTGAGTCGTATTAAAAAA	
CRISPR/Cas	Universal for three genes	ROX-dT15-BHQ2	ROX-TTTTTTTTTTTTTTT-BHQ2	

Table S3. Characterization of target genes.

Feature	Gene 1 (LAMP)	Gene 2 (RPA)	Gene 3 (RPA)
Length of amplified region	227	450	157
GC%	42	54	33
Tandem repeats (>=4)	1 (TCAT)	0	1 (ATTT)
Poly(GC)$_2$ (higher Z DNA fold)	0	0	0
Palindrome	5 sites (6 bp)	5 sites (6 bp)	2 sites (6 bp)
poly site (>=5)	2 A(T) site (5 bp)	A(T) site (6 bp), G(C) site (5 bp)	A(T) site (6 bp), A(T) site (5 bp)
G-quadruplex structure	No	No	No

Section 2. Preparation of gold nanoparticles and conjugate of gold nanoparticles with antibodies

Gold nanoparticles (GNPs) were synthesized using the Frens method [1], with slight changes. One milliliter of 1% $HAuCl_4$ was added to 95 mL of deionized water. The mixture was continuously stirred and heated to the boiling point; then, 4 mL of 1% sodium citrate was added. The GNP solution was continuously boiled for another 30 min, then cooled and stored at 4 ◦C for future use.

Conjugation of GNPs with mouse monoclonal antibodies specific to fluorescein (anti-FAM) was prepared as described by Safenkova et al. [2]. The solution of GNPs was adjusted to pH 9.5. Then, anti-FAM antibodies were added at a ratio equal to 12 µg per 1 mL of GNPs solution. The synthesis was carried out at RT for 1 h, with continuous mixing using a shaker. BSA as a blocking reagent was added to reach a final concentration of 0.25%. The mixture was centrifuged at 15,000× g for 30 min to separate the Au NP conjugates. Afterwards, the synthesized conjugates were resuspended in conjugate buffer (10 mM Tris buffer, pH 7.4, containing 0.25% BSA, 0.05% Tween 20 and 1% sucrose).

The hydrodynamic sizes of the nanoparticles, their conjugates and conjugate buffer were measured using Zetasizer Nano (Malvern Panalytical, Malvern, UK). All measurements were performed at 25 °C, and scattering angle was equal to 173 °C. Obtained distributions of hydrodynamic diameters are presented in Figure S1.

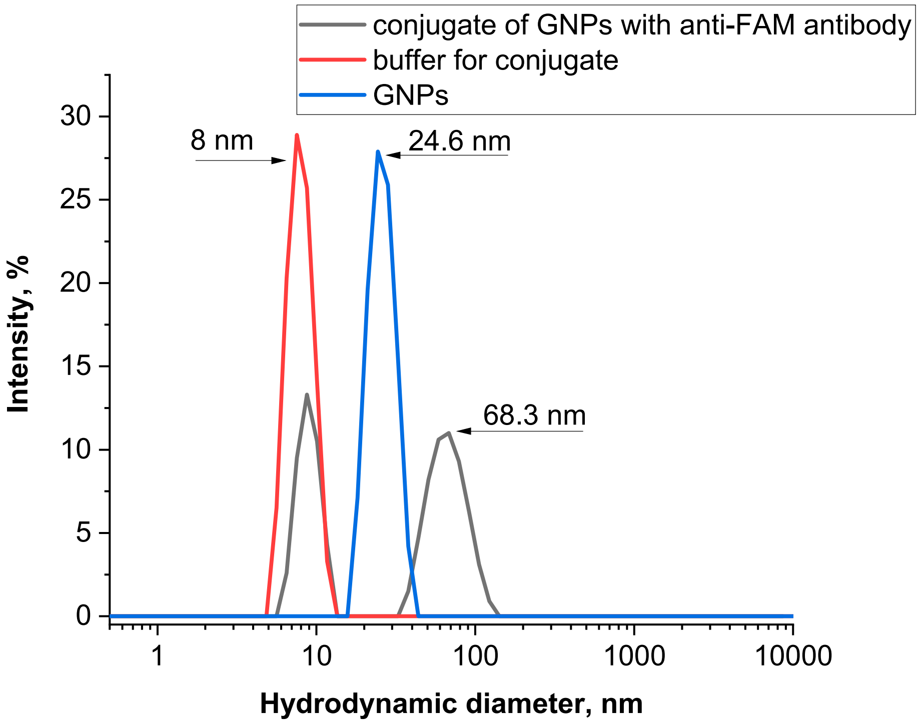

Figure S1. – Distributions of hydrodynamic diameters of gold nanoparticles, their conjugates with anti-FAM antibodies, and conjugate buffer obtained through DLS. Numbers indicate average hydrodynamic diameters for each sample.

1. Frens, G., Controlled Nucleation for the Regulation of the Particle Size in Monodisperse Gold Suspensions. Nature Physical Science 1973, 241, 20.
2. Safenkova, I. V.; Ivanov, A. V.; Slutskaya, E. S.; Samokhvalov, A. V.; Zherdev, A. V.; Dzantiev, B. B., Key significance of DNA-target size in lateral flow assay coupled with recombinase polymerase amplification. Anal. Chim. Acta 2020, 1102, 109-118.

Section 3. Characterization of DNA fragments of target genes obtained from *E. amylovora*

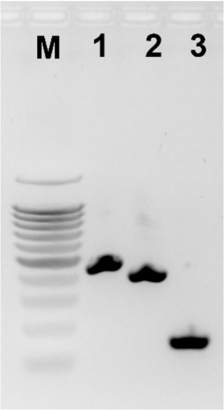

Figure S2. – Fragments of target genes after electrophoresis in 2% agarose gel. 1 - recombinase A, 2. phosphoribosyl transferase, 3. AMY1267.

Section 4. Comparison of test strips after testing LAMP products using all combination of labeled primers

Table S4. Comparison of test strips after testing LAMP products after amplification of phosphoribosyl transferase gene (gene-1) using all combinations of labeled primers.

	F3-b	B3-b	FIP-b	BIP-b	FL-b	BL-b
F3-F	**ND***	**ND**	**ND**	**ND**	**ND**	**ND**
B3-F	**ND**	**ND**	**ND**	**ND**	**ND**	**ND**
FIP-F	**ND**	**ND**	**ND**	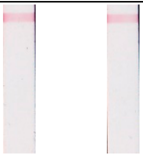 - +	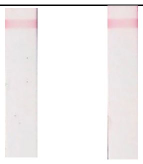 - +	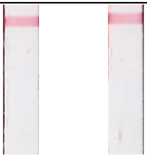 - +
BIP-F	**ND**	**ND**	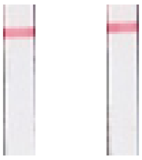 - +	**ND**	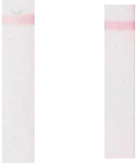 - +	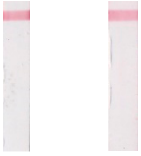 - +
FL-F	**ND**	**ND**	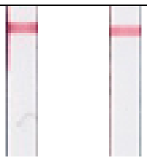 - +	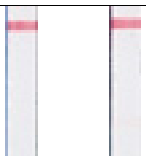 - +	**ND**	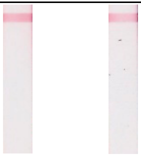 - +
BL-F	**ND**	**ND**	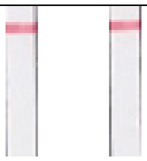 - +	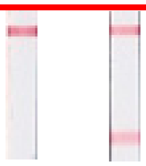 - +	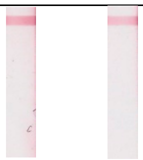 - +	**ND**

***ND** – no data. The combination was not tested, as the labeled product is expected to be minor or not possible at all.

Section 5. Cross-reactivity testing

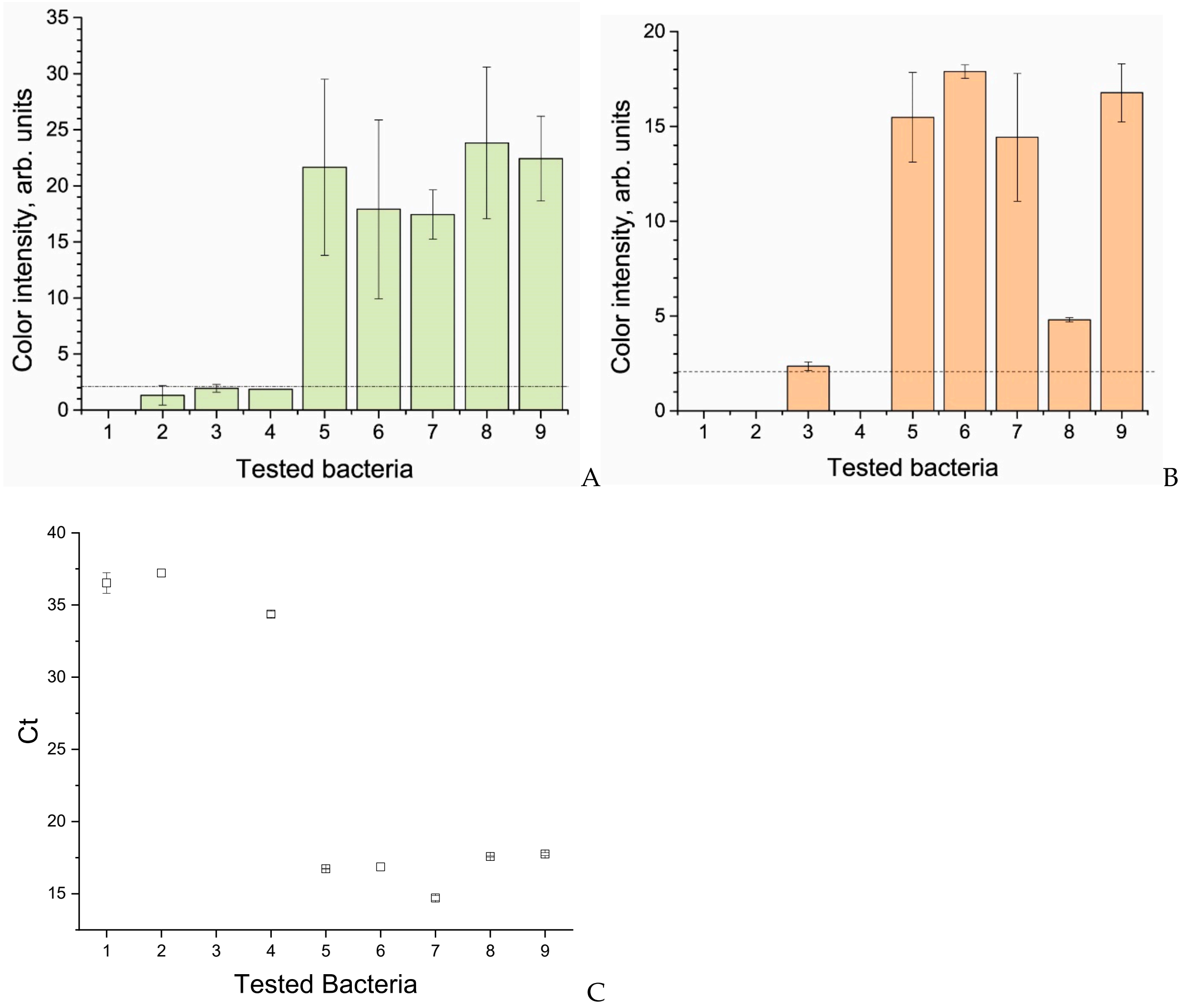

Figure S3. – Testing bacterial pathogens with RPA (A), LAMP (B), and PCR (C). 1 – *D. solani*, 2 – *R. solanacearum*, 3 – *P. atrosepticum*, 4 – *C. michiganensis* subsp *michiganensis*,.5 – VNIIKR KKE 3, 6 – VNIIKR FEa14, 7 – ACW56400, 8 – VNIIKR TE 16, 9 – VNIIKR KE 52.

Section 6. Spearman correlations of data for *E. amylovora* detection by five methods

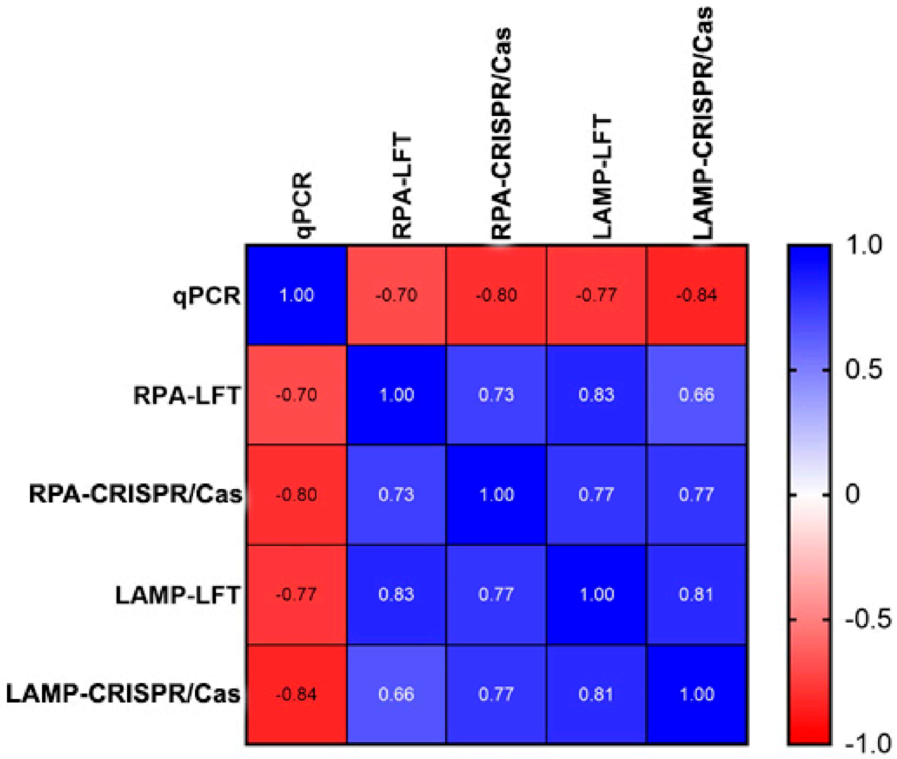

Figure S4. – Spearman correlation coefficients for data obtained at the testing samples from plant leaves (these data are presented in Figure 5) by qPCR, RPA-LFT, LAMP-LFT, RPA-CRISPR/Cas, and LAMP-CRISPR/Cas.
